# Medical schools producing the most physical medicine and rehabilitation residents: An analysis of matriculating residents from 2017 to 2021

**DOI:** 10.1002/pmrj.13216

**Published:** 2024-07-18

**Authors:** Devon T. Shannon, Paige M. Chase, Bailey W. Frei, Trevor Anesi, Aaron J. Yang

**Affiliations:** ^1^ Department of Physical Medicine and Rehabilitation Vanderbilt University Medical Center Nashville Tennessee USA; ^2^ Vanderbilt University School of Medicine Nashville Tennessee USA

## Abstract

**Background:**

Residency choice is often influenced by experiences in medical school. It is unclear what potential factors contribute to medical schools producing higher numbers of physical medicine & rehabilitation (PM&R) residents.

**Objective:**

To identify the medical schools producing the most PM&R residents from 2017 to 2021 and potential influencing factors toward this production.

**Design:**

Descriptive, cross‐sectional study.

**Setting:**

Accreditation Council for Graduate Medical Education accredited PM&R programs; allopathic/osteopathic/international medical schools.

**Interventions:**

REDCap Survey.

**Participants:**

Representatives from medical schools producing the most PM&R residents.

**Methods:**

The medical schools that produced the most PM&R residents from 2017 to 2021 were identified using publicly available information on the internet. A subgroup of the highest producing schools were surveyed to determine potential factors that contributed to production of PM&R residents.

**Main Outcome Measure:**

Medical schools with the highest number of matriculated PM&R residents from 2017 to 2021; potential factors influencing matriculating PM&R residents.

**Results:**

The medical school that produced the most PM&R residents from 2017 to 2021 was New York Institute of Technology College of Osteopathic Medicine. Nine of the 11 medical schools producing the most PM&R residents were osteopathic. Of osteopathic graduates applying to residency, 2.87% matriculated into PM&R residencies compared to 1.21% of allopathic graduates (*p* < .001), though a greater number of allopathic graduates overall were represented. Among survey respondents 93.3% (14/15) attributed exposure to PM&R faculty/residents and exposure to PM&R through medical school curriculum as perceived factors contributing to production of PM&R residents.

**Conclusion:**

Osteopathic medical schools accounted for most of the schools producing the highest number of PM&R residents. A statistically significant higher percentage of osteopathic graduates were found to pursue PM&R as a career compared to allopathic counterparts although the total number of students entering PM&R was greater from allopathic schools. Potential factors contributing to medical students pursuing PM&R included faculty/resident involvement with medical students, and PM&R exposure through curriculum or interest groups.

## INTRODUCTION

The decision of which specialty to pursue may be one of the most important decisions a medical student will make. It is often thought that there are identifiable experiences, such as shadowing or rotations, a student has prior to or during their medical school career that lead to the decision of pursuing residency within the field of physical medicine and rehabilitation (PM&R).^1^ As PM&R is a growing field, it is important to identify reproducible factors that can lead students to pursue a residency in PM&R to encourage continued growth of the field.^2^ A review of current studies indicated that early exposure to PM&R may be a vital factor to increasing interest in the specialty.^3^, ^4^ Neurosurgery and urology have published studies of which medical schools produce the most residents in their respective fields, in addition to identifying factors these schools displayed to explain these trends. Identifiable factors included the presence of a home residency program, number of clinical faculty, research funding, presence of interest groups, and rankings on national ranking metrics.^5^, ^6^ These highlighted studies were used as a framework for this current study. A prior survey in the field of PM&R was performed in 1983, which identified three possible contributing factors to medical students choosing PM&R: medical school graduating class size, presence of a PM&R department in the medical school, and the presence of a PM&R residency program at the medical school.^7^ The goal of our study was to provide an updated list of medical schools that produced the highest number of PM&R residents from 2017 to 2021. The highest producing medical schools were then surveyed to identify any contributing factors to their high matriculation rates. Considering prior studies have identified the presence of a residency program at the medical school as a contributing factor toward specialty choice, we also looked at matriculation rates between allopathic and osteopathic medical schools, as most PM&R residency programs are associated with allopathic medical schools.

## METHODS

### Data collection

This project was reviewed by the Vanderbilt University Medical Center institutional review board and categorized as exempt. A list of Accreditation Council for Graduate Medical Education (ACGME) accredited PM&R programs was obtained via the Association of American Medical Colleges (AAMC) Electronic Residency Application Service (ERAS) Directory.^8^ Data concerning which medical schools and the number of individuals from each medical school represented at all ACGME accredited PM&R programs in the United States and Puerto Rico were collected from 2017 to 2021 (ie, residency graduating classes of 2021–2025). The data were collected via publicly available information on the internet including, but not limited to, residency program websites, medical school websites, Linked‐In, Doximity, and social media platforms. A deidentified database was created that logged all PM&R programs and the medical schools represented by residents at those programs over the study period. Medical schools, and the number of residents represented by those medical schools, were then tallied to determine which schools were most represented.

**FIGURE 1:**
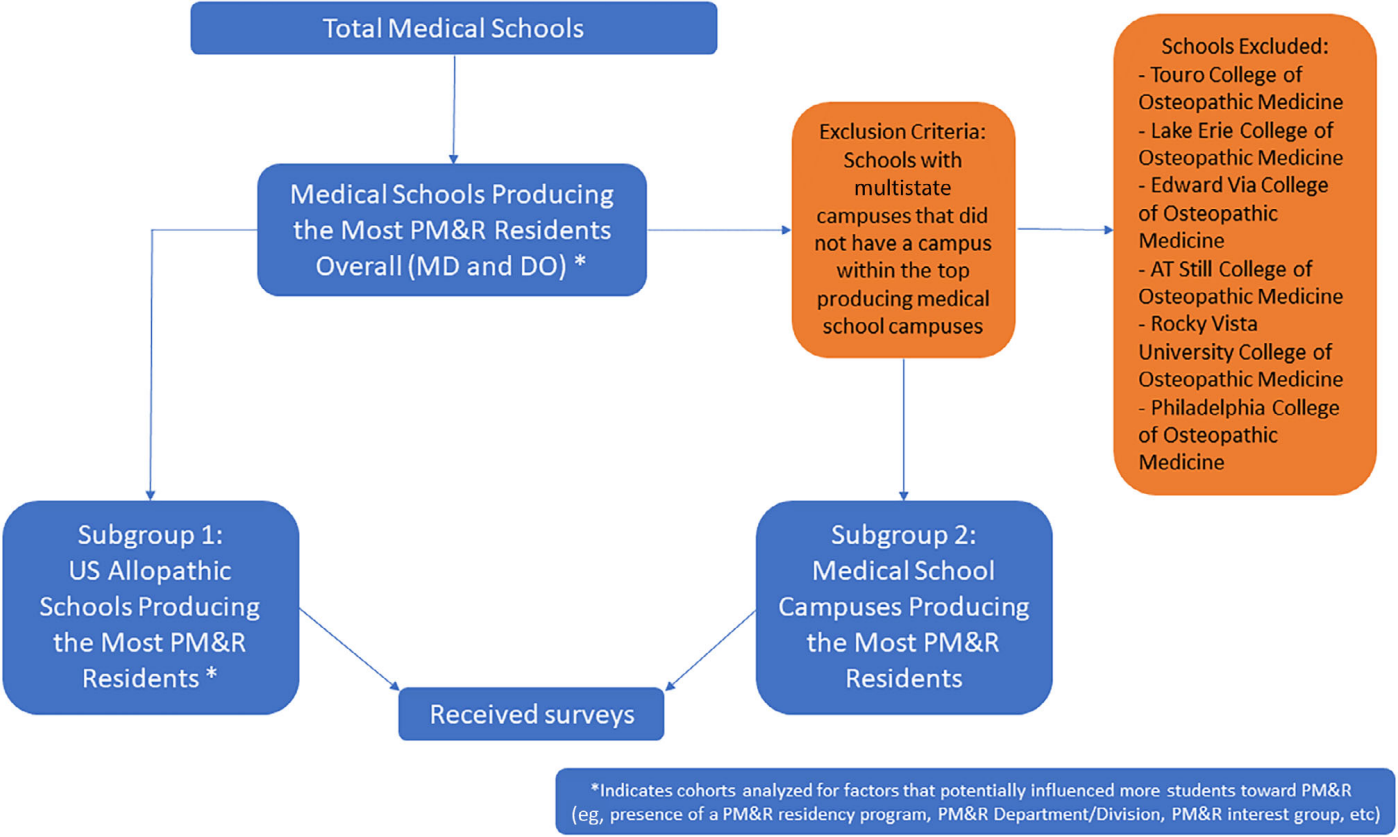
Subgroups identified from list of medical schools producing the most PM&R residents. PM&R, physical medicine and rehabilitation.

After the list of medical schools producing the most PM&R residents was generated, two additional subgroups were identified as some osteopathic medical schools had multistate campuses and publicly available information could not discern which campuses matriculated more PM&R residents than others (Figure 1). The first subgroup represented the U.S. allopathic medical schools that produced the most PM&R residents. This group was specifically created to analyze intrinsic factors that may have influenced students to PM&R, such as home residency program and Doximity rankings, and look for differences between this subgroup and the main group of top producing medical schools overall. The second subgroup represented the specific medical school campuses (allopathic and osteopathic) that produced the most PM&R residents in which we could identify one medical campus producing the most PM&R residents, as multistate campuses may not display the same factors that lead students to pursue PM&R residencies. We did include medical schools with multiple campuses in the same state in the second subgroup and did not differentiate campuses in such instances.

The overall list of medical schools producing the most PM&R residents, including subgroup one, was analyzed to determine if the medical schools had a home PM&R residency program (information obtained via ERAS website),^8^ a PM&R interest group (information via Association of Academic Physiatrists [AAP] database),^9^ an affiliation with a top 10 rehabilitation hospital according to U.S. News & World Report (USNWR),^10^ and a top 10 ranking of home residency program via Doximity rankings.^11^

Data were also obtained from the National Resident Matching Program (NRMP) main residency match database from 2017 to 2021 to determine the number of students who applied to any residency program and those who matched into PM&R.^12^ This was done by calculating the number of residents who matched into either categorical or advanced PM&R programs over the study time frame from allopathic (MD) or osteopathic (DO) medical schools and by summating the total number of residency applicants over the study period. This study also sought to determine the relative percentage of applicants who matriculated into PM&R residencies relative to any residency program with the goal of seeing if there was any statistically significant difference between allopathic versus osteopathic medical schools.

### Survey data

Subgroups one and two were included in the survey portion of the study as seen in Figure 1. A list of these schools can be found in Table 1. A qualified representative (dean, residency program director, director of clinical education, or residency counselor) at the schools was emailed a deidentified REDCap survey to determine factors thought to contribute to these schools producing more PM&R residents over the study period. The survey was limited to five multiple choice questions and two free response questions to minimize the time needed to respond and maximize response rates (Table 2). Email reminders were sent on three separate occasions to maximize survey responses. Study data were collected, stored, and analyzed within the REDCap electronic data capture tools hosted at Vanderbilt University Medical Center.^13^, ^14^

**TABLE 1:** List of medical schools that received surveys.

U.S. allopathic medical schools producing the most PM&R residents (subgroup one)
University of Toledo College of Medicine and Life Sciences^a^
Rutgers New Jersey Medical School^a^
Louisiana State University School of Medicine
Indiana University School of Medicine
Michigan State University College of Human Medicine
University of Texas‐Houston, McGovern Medical School
Loma Linda University School of Medicine
University of Cincinnati College of Medicine
Virginia Commonwealth University School of Medicine
Penn State College of Medicine
Rutgers Robert Wood Johnson Medical School
Wayne State University School of Medicine

Medical school campuses producing the most PM&R residents (subgroup two)
New York Institute of Technology College of Osteopathic Medicine—Long Island Campus (DO)
University of North Texas—Texas College of Osteopathic Medicine (DO)
University of Toledo College of Medicine and Life Sciences (MD)
Michigan State University College of Osteopathic Medicine (DO)
Nova Southeastern University College of Osteopathic Medicine (DO)
Rutgers New Jersey Medical School (MD)
St Georges University School of Medicine (International‐MD)
Rowan University School of Osteopathic Medicine (DO)
Lincoln Memorial University‐DeBusk College of Osteopathic Medicine (DO)
Western University—College of Osteopathic Medicine of the Pacific, California Campus (DO)
Kansas City University College of Osteopathic Medicine (DO)
Midwestern University—Chicago College of Osteopathic Medicine (DO)

Abbreviation: PM&R, physical medicine and rehabilitation.

^a^ Received only one survey.

**TABLE 2:** List of REDCap survey questions.

1. Does your medical school offer clinical rotations in PM&R?
Yes
b No
c Unknown
2. Does your medical school have a required rotation within PM&R?
Yes
b No
c Unknown
d No rotations offered
3. What training year does your medical school offer rotations within PM&R? (Select all that apply)
First year
b Second year
c Third year
d Fourth year
e No rotations offered
4. Of the events listed, which do faculty members or residents within the field of PM&R (either within the PM&R department at your medical school or in the community) participate in at your medical school? (Select all that apply)
Lectures
b Interest group meetings
c Ultrasound workshops
d Career advising
e Other
f None
5. If selected “other” above, please describe PM&R faculty/resident involvement within the medical school.
Free text
6. What factors do you think have contributed to students from your medical school pursuing a residency within PM&R? (Select all that apply)
Faculty and resident exposure
b Exposure to PM&R during medical school curriculum (lectures, rotations, etc)
c Interest group meetings
d Research exposure within PM&R
e Having PM&R faculty members serving within the medical school leadership
f Other
7. If selected “other” above, please explain what other factors you believe contribute to medical students at your medical school pursuing a residency within PM&R.
Free text

Abbreviation: PM&R, physical medicine and rehabilitation.

### Statistical analysis

Statistical analysis was run using the Stata/BE 17.0 program. A two‐sample proportion test using variables was run to compare the percentage of allopathic versus osteopathic medical students that matriculated into PM&R. The two‐sample proportion test determined whether there would be no difference in percentage between allopathic and osteopathic medical students matriculating into PM&R. Furthermore, values were assigned as “0” if a student matched into something other than PM&R or “1” if the student matched into PM&R, allowing for the proper performance of the two‐sample proportion test.

## RESULTS

### Residency/medical school demographics

According to the AAMC ERAS directory, there were 106 PM&R residency programs eligible to participate in the residency match at the time of this study.^8^ A program was excluded if it did not have residents in any years of the study period, primarily indicating they were newer programs established after 2021. Eight programs were excluded, leaving 98 programs for analysis. According to the NRMP data from 2017 to 2021, there were 2303 individuals who matched into PM&R residencies. Using publicly available data, this study captured 2082 individuals who matched into PM&R programs from the 98 PM&R residency programs, accounting for approximately 90.4% of the NRMP data over this period. Out of these 2082 total individuals, 55.6% were U.S. allopathic graduates (including Puerto Rico) (1158 individuals), 36.8% were U.S. osteopathic graduates (767 individuals), and 7.5% were international graduates (157 individuals). There were a total of 149 U.S. allopathic (including Puerto Rico), 32 osteopathic, and 65 international medical schools represented.

According to NRMP data from 2017 to 2021, there were 95,474 U.S. allopathic seniors and 26,770 osteopathic seniors who applied for residencies over that period. Based on our data, 1.20% of U.S. allopathic applicants from 2017 to 2021 matriculated into PM&R residencies, compared to 2.87% of osteopathic applicants (Table 3). There was a statistically significant difference between U.S. allopathic and osteopathic PM&R matriculation rates, with a higher rate of osteopathic students matriculating into PM&R residencies compared to allopathic students (*p* < .001) (Table 3).

**TABLE 3:** Matriculation rates into PM&R residencies between allopathic and osteopathic medical schools from 2017 to 2021 when comparing study results to national data from NRMP.

Type of medical school	Number of PM&R residents	Number of applicants in NRMP match	Matriculation rate into PM&R^a^
Allopathic (MD)	1158	95 474	1.21%
Osteopathic (DO)	767	26 770	2.87%

Abbreviations: NRMP National Resident Matching Program; PM&R, physical medicine and rehabilitation.

^a^ Statistically significant difference between U.S. allopathic and osteopathic matriculation rates with a higher rate of osteopathic students matriculating into PM&R residencies compared to allopathic students (*p* < .001).

### Medical schools producing the most PM&R residents

**FIGURE 2:**
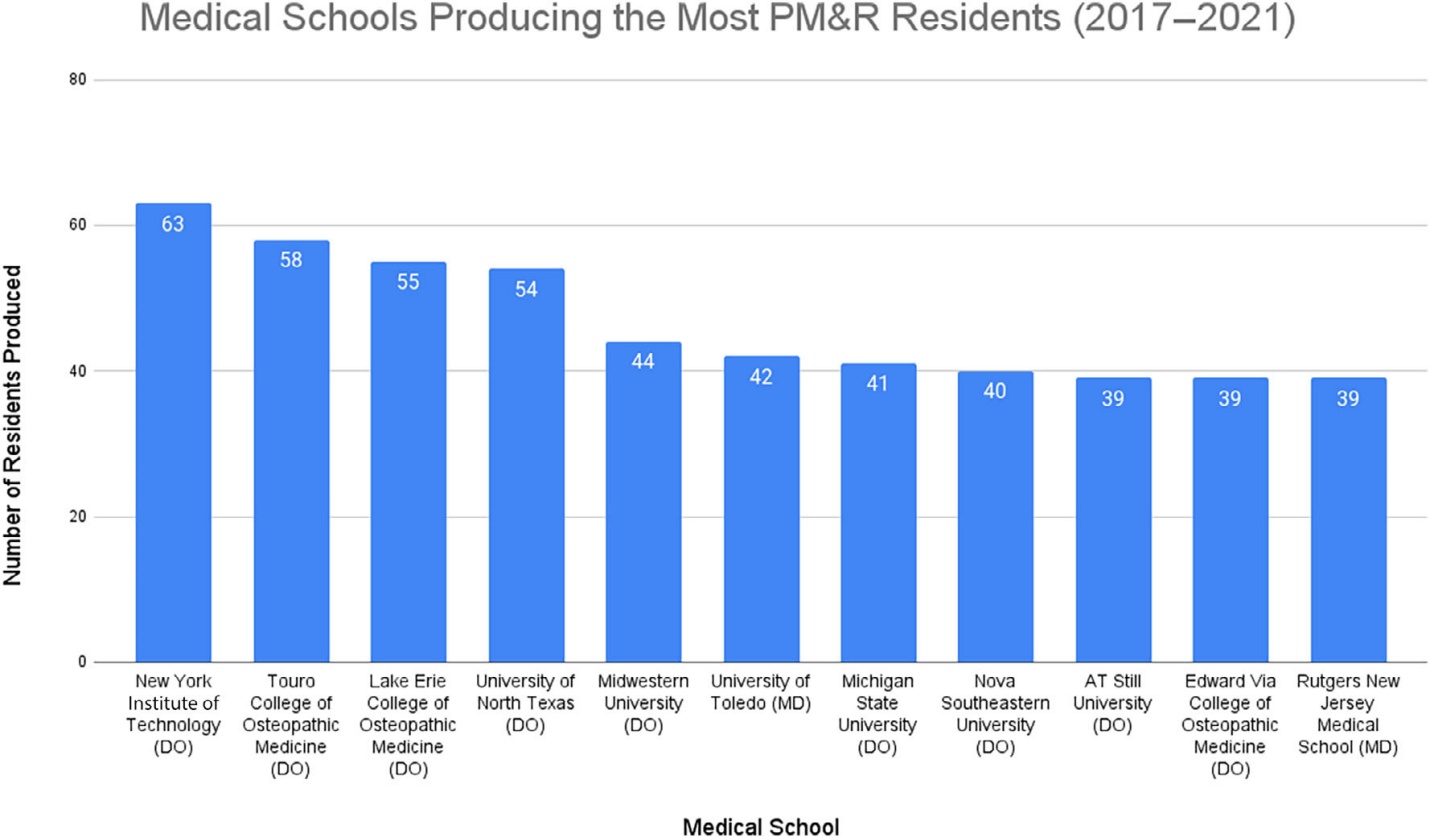
Medical schools producing the most PM&R residents from 2017 to 2021. PM&R, physical medicine and rehabilitation.

Figure 2 shows the medical schools that produced the most PM&R residents. Nine of the 11 schools (81.8%) that produced the most PM&R residents were osteopathic medical schools. The medical school producing the most PM&R residents from 2017 to 2021 was New York Institute of Technology College of Osteopathic Medicine (DO) with 63 residents (Figure 2). Only 27.3% (3/11) of the schools had a home PM&R residency program and department/division associated with any campus (University of Toledo, Michigan State University [DO], and Rutgers New Jersey Medical School). According to AAP website data, the top six schools all had a PM&R interest group registered with the AAP at any of their campuses. In addition, only one school (Rutgers New Jersey Medical School) had a PM&R residency program ranked in the top 10 on Doximity or a rehabilitation hospital affiliate ranked in the top 10 on USNWR.

### U.S. allopathic medical schools producing the most PM&R residents

**FIGURE 3:**
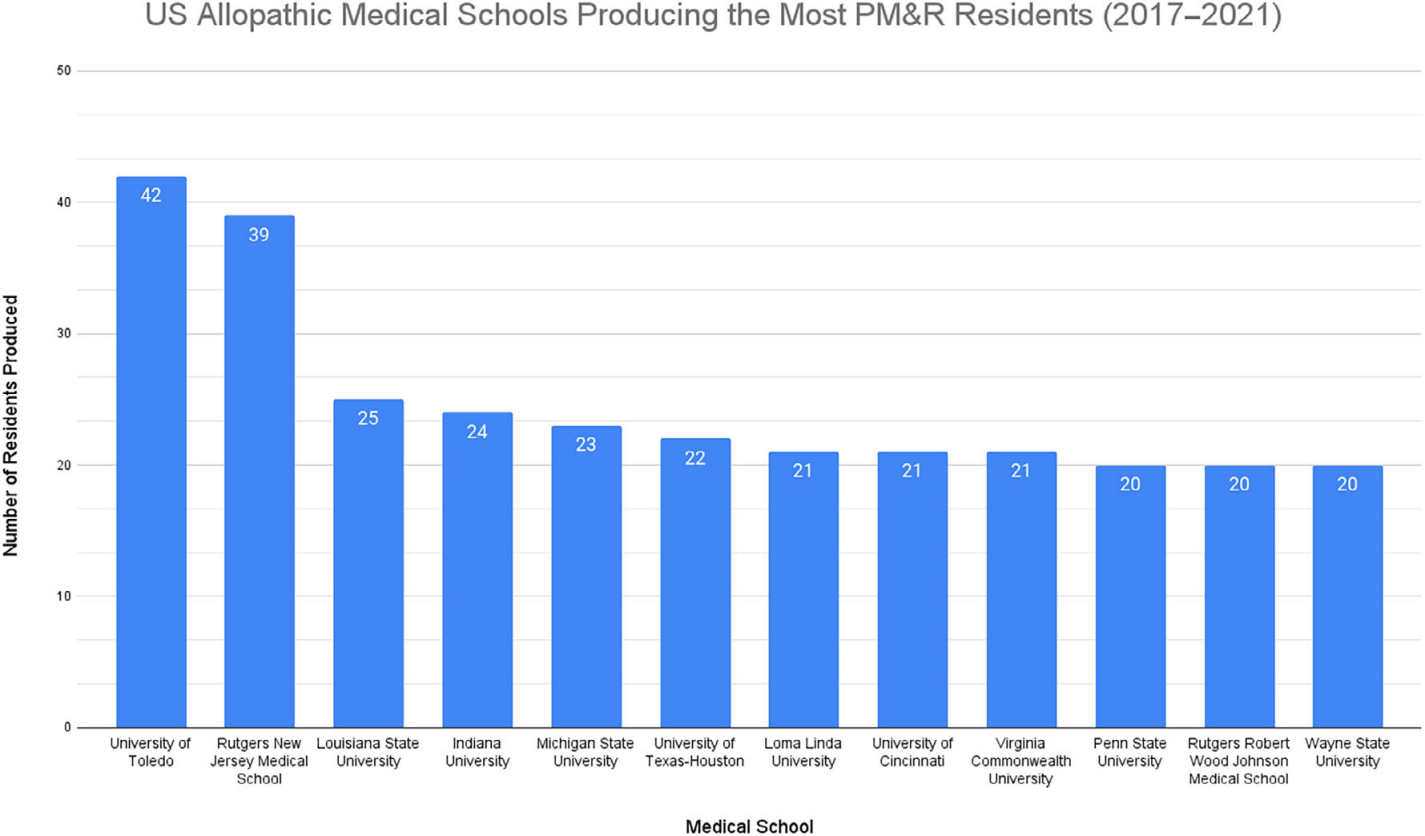
U.S. allopathic medical schools producing the most PM&R residents from 2017 to 2021. PM&R, physical medicine and rehabilitation.

Figure 3 shows the U.S. allopathic medical schools that produced the most PM&R residents. All the U.S. allopathic schools had home PM&R residency programs and departments/divisions (including Michigan State University's allopathic program as having a home program given affiliation with the osteopathic program). Only two schools had a top 10 ranking on USNWR and Doximity with regard to ranking of rehabilitation hospitals and PM&R residency programs, respectively (Rutgers New Jersey Medical School and University of Texas‐Houston). According to AAP website data, 75% (9/12) of the schools had a PM&R interest group registered with the AAP.

### Survey results

**FIGURE 4:**
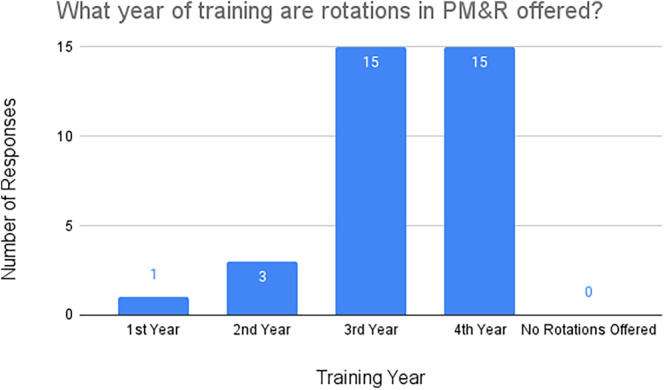
Years of training in which rotations are offered within PM&R at survey respondents' medical schools (n = 15). PM&R, physical medicine and rehabilitation.

A total of 22 surveys were sent to the medical schools represented by subgroups one and two (Table 1). Although the University of Toledo and Rutgers New Jersey Medical School were in both subgroups, they each received one survey. A total of 68.2% (15/22) of responses were collected. Out of the respondents, all the medical schools reported they offered rotations within PM&R during students' third and fourth years of medical school. Only 20.0% (3/15) of respondents stated their school offered rotations in the second year and one offered rotations in the first year (Figure 4). Additionally, 20.0% (3/15) of respondents indicated their medical school has a required rotation within PM&R.

**FIGURE 5:**
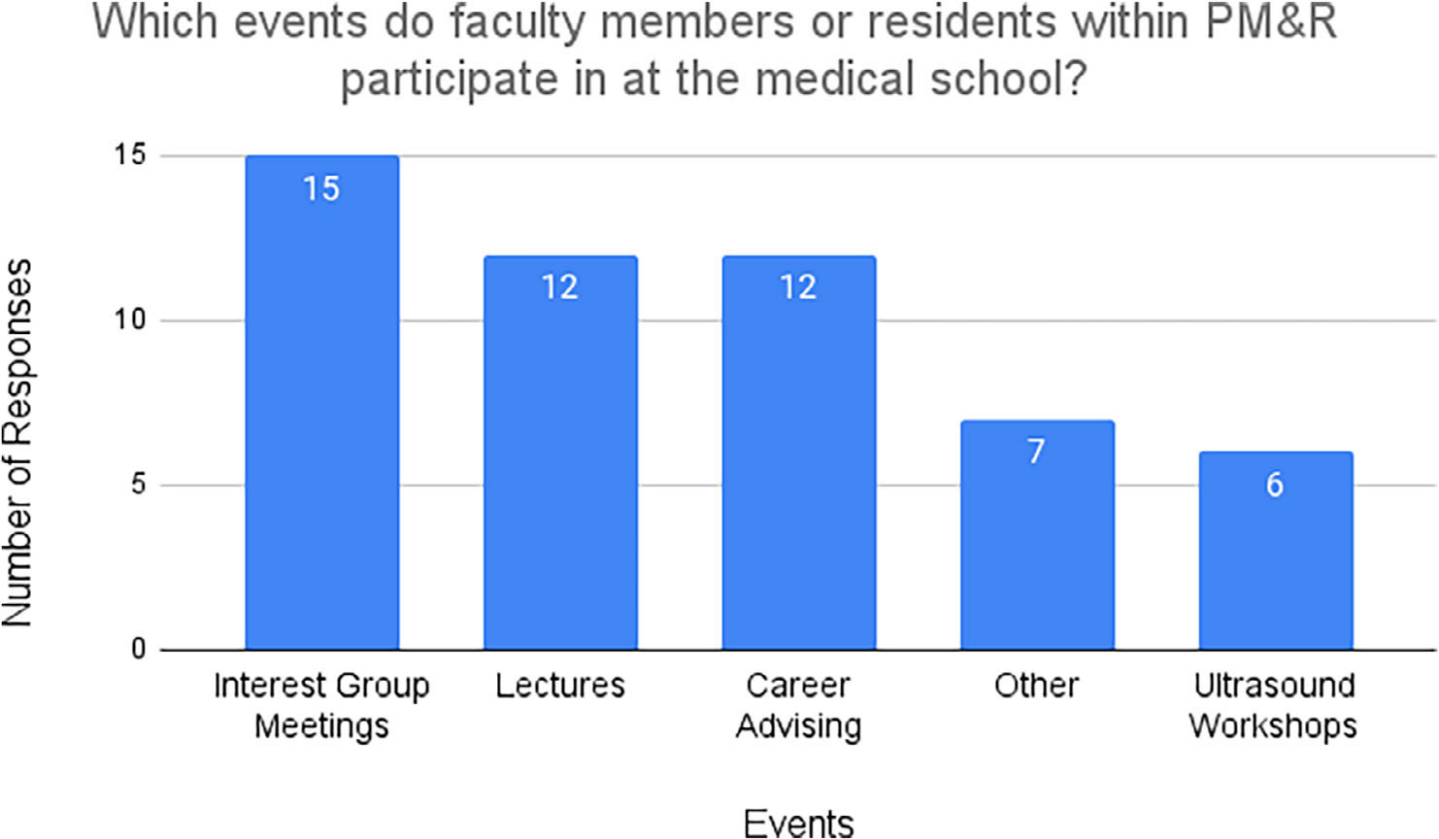
Events in which faculty/residents within PM&R participate at survey respondents' medical schools (n = 15). PM&R, physical medicine and rehabilitation.

**FIGURE 6:**
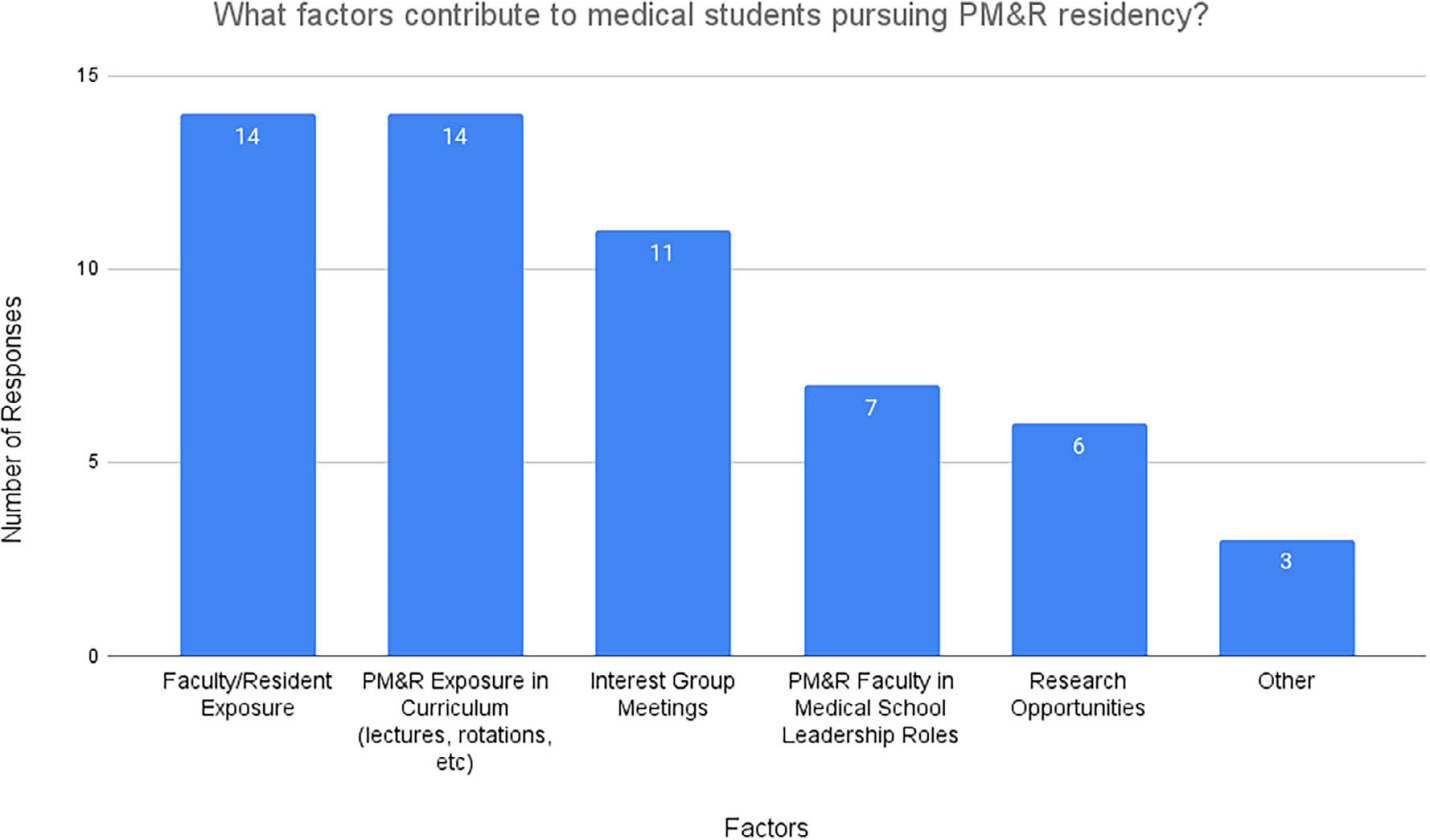
Factors perceived by survey respondents to be influencing students' decision to pursue a residency in PM&R (n = 15). PM&R, physical medicine and rehabilitation.

With regard to what events faculty or residents within PM&R participate in at the medical schools, all respondents listed interest groups as a means of participation, 80.0% (12/15) listed lectures within the medical school and career advising as other methods of participation (Figure 5), and 40.0% (6/15) listed ultrasound workshops as a way PM&R faculty/residents participate within the medical school. Free responses were also gathered with regard to avenues PM&R faculty/residents interacted with medical students and comments included participation with physical exam workshops, anatomy labs, career fairs, pain/disability workshops, and medical school life groups (Table 4). In terms of determining which perceived factors survey respondents felt contributed to students pursuing PM&R, 93.3% (14/15) attributed this to faculty/resident exposure and exposure to the specialty through medical school curriculum, be it through lectures, rotations, or other means (Figure 6). Interest group meetings and PM&R faculty in medical school leadership roles were other influential factors noted on surveys. Free responses were also gathered and multiple respondents mentioned their school's osteopathic mission as a perceived factor influencing students to PM&R as they felt it closely tied with the specialty (Table 4).

**TABLE 4:** Free response answers to survey questions.

Free responses answers to the prompt of “please describe PM&R faculty/resident involvement within the medical school”
“Clinical correlation for anatomy course for first‐year medical students. Musculoskeletal exam workshop for second‐year medical students”
“Physical examination courses”
“Research opportunities”
“Teaching physical exam skills”
“Life Community Groups: longitudinal small groups that meet weekly for 4 years of medical school with a faculty mentor for connection and support. We have 2 core faculty who are mentors for this.”
“Career Fairs”
“1. Pain and Disability Day workshop. 2. Medical student fair/workshop experience. 3. Research Projects”

Free response answers to the prompt of “please explain what other factors you believe contribute to medical students at your medical school pursuing a residency within PM&R"
“We have had good success in students choosing PM&R over the past decade. In that time period we have had close to 100 students choose PM&R. With such a high number word of mouth has spread which I believe created interest each year.”
“We are a large osteopathic medical school. There are several areas of overlap in skills and competencies within the required (additional) OMM curriculum for osteopathic medical students and the field of PM&R.”
“We are an osteopathic medical school and it is a natural field for MSK and OMM.”
“Involvement of medical students into resident community events.”

Abbreviations: MSK, musculoskeletal; OMM, osteopathic manipulative medicine; PM&R, physical medicine and rehabilitation.

## DISCUSSION

The majority of medical schools producing the highest number of PM&R residents from 2017 to 2021 were osteopathic, accounting for nine of the 11 top medical schools with the highest number of medical school graduates entering PM&R. In addition, over this 5‐year time frame, there was a higher rate of osteopathic medical students matriculating into PM&R compared to allopathic students when looking at applicants to all medical specialties. However, there were still more U.S. allopathic graduates who entered PM&R (1158 vs. 767) considering there were far more U.S. allopathic medical schools (149 vs. 32) accounted for in our study's data. Prior studies have shown that the presence of a home residency program can influence the decision of medical students choosing a particular field.^5^, ^6^ However, our study demonstrated different findings in that out of the top 11 medical schools, only three had a home residency program and/or a PM&R department/division. The presence of an interest group may have more profound impact as the top six medical schools all had a PM&R interest group. Interestingly, national reputation such as ranking in the top 10 on Doximity or rehabilitation affiliate ranked in the top 10 on USNWR did not appear to significantly affect medical students entering PM&R.

Looking specifically at the top U.S. allopathic medical schools matriculating students into PM&R, all the medical schools had a home PM&R residency program and a PM&R department/division and 75% of the schools had a PM&R interest group identified. Similar to the trend seen with the overall osteopathic and allopathic list, only two schools had a top 10 ranking on USNWR and Doximity.

When looking at specific influencing factors from survey responses received from medical schools, a few trends stood out. All the medical schools offered rotations within PM&R during the third and fourth years whereas only 20% offered rotations in the second year and only one school offered any exposure during first year. Only 20% indicated a required rotation in PM&R. Required PM&R rotations could be a way to increase exposure to the field and have been shown in prior studies to increase students' knowledge of and interest in PM&R.^15^, ^16^, ^17^ In addition, early exposure to PM&R has been shown to be a crucial factor influencing medical students to pursue a PM&R residency.^4^

Aside from rotations, PM&R participation at the medical school level is an important suggested way to provide exposure to medical students. Interest groups, lectures, and career advising were the top three ways survey respondents mentioned how students got connected to PM&R. Most of the respondents also identified faculty and resident exposure as important factors.

A possible area for PM&R integration is within a medical school's musculoskeletal education, as previous studies have shown that musculoskeletal workshops can increase understanding of the role of PM&R and foster interest in pursuing a career in the field.^18^ Musculoskeletal education appears to be an area the schools in this study have accessed as multiple survey respondents cited physical examination teaching or anatomy courses as ways they taught within the medical school. This is consistent with past attempts within the field of PM&R to integrate within medical school's anatomy and physical exam curricula.^19^, ^20^, ^21^, ^22^ Ultrasound courses were also noted by survey respondents as a way PM&R was introduced to medical students, which is supported by a prior study that showed ultrasound workshops can effectively increase medical student knowledge about the field.^23^ Another opportunity identified by survey respondents to capture students' interest was through career fairs. This falls in line with a prior study that found career fairs within the field of PM&R can increase students' knowledge of and interest in PM&R, and can be an opportunity for PM&R programs to invite students from medical schools outside their home school to get exposure to the specialty.^24^ These are only a few ways for increased PM&R exposure for medical students; more research needs to be done on the most effective ways to garner students' interest and to integrate the specialty into medical education.

Lastly, the AAMC publishes a graduation questionnaire that looks into the factors that may influence graduating medical students to choose a certain specialty. Although not specific to PM&R and focused on allopathic graduates, some factors stood out as being a strong influence on choosing a specialty including role model influence, work/life balance, fit with personality, and content of specialty.^25^ Future directions for research include looking at PM&R specific data on specialty decision making as well as a focus on factors that influence osteopathic medical students to choose PM&R, considering our top medical schools producing the most PM&R residents were osteopathic.

## LIMITATIONS

There were several limitations to this study. The largest limitation was the inability to determine the specific campus location for all residents in the study who attended a medical school with campuses across multiple states. This limited the ability to determine if certain medical school campuses were producing the most residents and if there were any campus‐specific factors that led to the production of more PM&R residents. This solely excluded osteopathic medical schools and led to inclusion of one international school in the survey analysis. This exclusion criterion was necessary as the curricula and PM&R exposure may differ at each medical school campus, making it unclear which campus and respective designee should receive the survey to determine the trends that their school had for producing PM&R residents.

To calculate the number of total applicants for residency programs from 2017 to 2021, only seniors from U.S. allopathic and osteopathic schools participating in the NRMP match data over that period were included. This excluded any nontraditional graduates (who applied outside of their senior year of medical school) entering the match in the designated time frame. This may make the rate of matriculation into PM&R appear higher than it is, whereas if those individuals were included, it would have increased the total number of applicants. Notably, this should not have altered the identified difference between allopathic and osteopathic schools regarding matriculation rates. Data were analyzed only with regard to applicants who matched into PM&R compared to the total number of applicants over the study period. Therefore, the rate of successful match into PM&R was not calculated. Further studies would be useful to analyze the trends of nonmatched applicants.

Another limitation of this study was that all information was obtained via publicly available data for the study to be reproducible and to comply with institutional review board requirements for exemption. This limited the number of residents captured as study personnel could not reach out to programs to obtain missing information that could not be found via publicly available information. Additionally, the data were temporal in nature, based on when the data were made available on whatever website they were obtained from. This means that there was no way to ensure that all the residents captured in our study stayed at or completed training at the residency program identified. It assumed that they had been enrolled there at some point and the medical school they graduated from was identifiable.

Further limitation stemmed from the fact that survey data had to be deidentified and anonymous so the determination of what schools specifically were providing each identified resource to medical students could not be identified. Thus, further analysis regarding possible resource differences among osteopathic, allopathic, and international schools could not be identified.

## CONCLUSIONS

Based on the results from this study, osteopathic medical schools represent a majority of the medical schools producing the most PM&R residents in addition to having a significantly higher percentage of medical students entering PM&R as a medical specialty compared to allopathic medical graduates. However, there are still more allopathic medical graduates entering PM&R over the study time frame considering there are more U.S. allopathic medical schools. Consistent with prior research on factors influencing recruitment to PM&R, this study suggests that exposure to PM&R throughout medical school could contribute to higher production of PM&R residents, whether that be through interest group participation, lectures, or rotations. Interestingly, our study showed that the presence of a home PM&R program did not appear to be a factor in a school producing more PM&R residents in this study. More studies are needed looking at both allopathic and osteopathic medical schools and how PM&R specific exposures may influence specialty decision making.

## DISCLOSURE

None.
